# Blocking the LncRNA MALAT1/miR-224-5p/NLRP3 Axis Inhibits the Hippocampal Inflammatory Response in T2DM With OSA

**DOI:** 10.3389/fncel.2020.00097

**Published:** 2020-05-12

**Authors:** Ping Du, Jiahui Wang, Yelei Han, Jing Feng

**Affiliations:** Department of Respiratory and Critical Care Medicine, Tianjin Medical University General Hospital, Tianjin, China

**Keywords:** MALAT1, MiR-224-5p, inflammation, intermittent hypoxia, type 2 diabetes mellitus

## Abstract

Studies have shown that diabetes can cause cognitive dysfunction, and cognitive dysfunction in patients with diabetes combined with obstructive sleep apnea (OSA) is more severe. LncRNAs are known to be associated with type 2 diabetes mellitus (T2DM) with OSA. This study aimed to investigate the role and underlying mechanism of the lncRNA MALAT1/miR-224-5p/NLRP3 axis in T2DM with OSA. qRT-PCR was used to quantify the expression of MALAT1, miR-224-5p, and NLRP3 in brain tissues. NLRP3 expression was assessed by immunohistochemistry (IHC) and immunofluorescent labeling. The interaction involving MALAT1, miR-224-5p, and NLRP3 was evaluated by transfection. Western blotting was utilized to evaluate the expression levels of the pathway-related proteins NLRP3, caspase 1, tumor necrosis factor-α (TNF-α) and interleukin-1 β (IL-1β) both *in vitro* and *in vivo*. qRT-PCR was used to assess the mRNA expression levels of NLRP3, caspase 1, TNF-α and IL-1β both *in vitro* and *in vivo*. In brain tissues of T2DM with OSA, MALAT1 and NLRP3 were overexpressed, while miR-224-5p was downregulated, which was consistent with subsequent cell experiments. We screened the miRNAs that could bind to MALAT1 and NLRP3 by the StarBase database and the TargetScanMouse7.2 website. Our research showed that among these miRNAs, the level of miR-224-5p was most significantly negatively correlated with the levels of MALAT1 and NLRP3. Also, a firefly luciferase assay showed that miR-224-5p, which is a target of MALAT1, directly reduced the expression of the downstream protein NLRP3. Overexpression of miR-224-5p significantly inhibited the expression levels of NLRP3, caspase 1, TNF-α and IL-1β *in vitro*. MALAT1 promoted NLRP3 expression by acting as a competing endogenous RNA and sponging miR-224-5p. MiR-224-5p reduces microglial inflammation activation through the regulation of NLRP3 expression, which ultimately affected the NLRP3/IL-1β pathway in the hippocampus. This suggests that miR-224-5p may serve as a potential target for T2DM and OSA therapy.

## Introduction

Obstructive sleep apnea (OSA) is a common disease that is characterized by recurrent upper airway obstruction during sleep, resulting in intermittent hypoxia (IH; Franklin and Lindberg, 2015). OSA patients are considered to be a potential risk for developing metabolic syndrome (Basta and Vgontzas, 2007). A representative metabolic syndrome disease is type 2 diabetes mellitus (T2DM), which is closely related to obesity and is characterized by an abnormal increase in blood glucose levels (Mahlangu et al., 2020). With the increasing incidence of T2DM, the number of patients with T2DM complicated with OSA is also increasing year by year (Lukas et al., 2014). Studies have shown that diabetes can cause cognitive dysfunction in the brain, and cognitive dysfunction in patients with diabetes and OSA is more severe. IH is thought to play an important role in the pathological consequences of OSA in a variety of organs, most likely through enhanced oxidative stress and inflammation (Lavie, 2015; Xu et al., 2019). The main cause of cognitive dysfunction is the irreversible damage to neurons in the hippocampus of the brain (Hicks et al., 1993). The reason for the damage to neurons, except for the direct damage, is that a large number of the activated microglia release neurotoxic substances and cause further damage to neurons (Yan et al., 2019). The production and release of tumor necrosis factor-α (TNF-α) and interleukin-1 β (IL-1β) lead to neuronal apoptosis and brain damage (Zhao et al., 2015). Previously, our team studied the activation of glial cells in the hippocampus and released HMGB1 to act on neuronal cells, causing neuronal damage and resulting in learning and cognitive dysfunction (Shi et al., 2018). Our research aims to find ways to reduce the activation of glial cells in the brain to alleviate cognitive dysfunction in patients with diabetes and OSA.

Long noncoding RNAs (lncRNAs) are a research hotspot at home and abroad. They are RNA molecules greater than 200 nt with no ability to encode proteins, but can participate in numerous biological processes (Li M. M. et al., 2019). Antisense lncRNAs are the reverse complementary sequences of endogenous RNA, accounting for approximately 60% of lncRNAs and most long-chain noncoding transcriptomes (Chen et al., 2018). MALAT1 is a lncRNA that was first found in early-stage, non-small cell lung cancer as a predictive factor for metastasis (Ji et al., 2003). In the past few years, the main focus has been on the relationship between MALAT1 and the prevalence of cancer (Cheetham et al., 2013). Recent studies have shown that lncRNA MALAT1 is not only involved in tumorigenesis and progression but also affects the progression of other diseases (Mishra et al., 2019). Some researchers now find that it has a lot to do with the occurrence of chronic diseases, such as stroke (Cao et al., 2020) and T2DM (Liu et al., 2019). MALAT1 may play an important role in the progression of T2DM with OSA.

Studies have shown that lncRNAs can exert regulatory effects by acting on specific microRNAs (miRNAs). MiRNAs are a class of short (21–24 nucleotides) noncoding RNAs (Zanchi et al., 2017) that exist in almost all biological tissues and regulate gene expression by interfering with RNA translation (Baek et al., 2008). Changes in miRNA composition are associated with a variety of human diseases, including inflammation (Marques-Rocha et al., 2015) and cancer (Li J. et al., 2019). Studies have shown that miRNAs play a role in the inflammatory response of stroke (Cao et al., 2020).

However, the important role of MALAT1 and miRNAs in the occurrence of T2DM with OSA needs further study to find new treatment strategies to improve the health of patients with cognitive disorders. In this study, we evaluated the protective role of miR-224-5p in KKAy mice (a mouse model of spontaneous type 2 diabetes; Iwatsuka et al., 1970) and KKAy mice exposed to IH. We also explored whether blocking the MALAT1/miR-224-5p/NLRP3 axis in activated microglia can inhibit nerve cell damage and ultimately reduce brain injury to alleviate cognitive impairment.

## Materials and Methods

### Animals

Male C57BL/6J mice (C57 mice) and KKAy mice were purchased from HFK Bio-Technology Company Limited (Beijing, China). Mice were put in a 12-h light/12-h dark cycle at constant room temperature (23°C) with free access to autoclaved water and food. After 2 weeks of adaptation, C57 mice (12 weeks old, weight 27–31 g) were randomly divided into the control group (C57 group, *n* = 8) and the IH group (C57+IH group, *n* = 8), and KKAy mice (12 weeks old, weight 37–40 g) were assigned to the T2DM group (KK group, *n* = 8) and the T2DM+IH group (KK+IH group, *n* = 8). We have developed a gas delivery program in a computer language that can deliver specific gas to the exposure chamber to simulate IH (similar to OSA; Guo et al., 2019). The IH exposure method consisted of reducing the oxygen concentration supplied to the exposure chamber to 5% for 30 s in each IH exposure cycle and then restoring the oxygen concentration to 21% for 90 s at a cycle rate of 30 cycles per hour for 8 h per day. The C57+IH group and the KK+IH group were exposed to IH for 4 weeks. For the intermittent normoxic air method for the C57 group and the KK group, the O2 concentration in the exposure chamber was maintained at 21% for 8 h/per day for 4 weeks. All animal experiments were approved by the Animal Ethics Committee of Tianjin Medical University and were carried out as required.

### Cell Culture

The microglial BV2 mouse cell line (National Infrastructure of Cell Line Resource, Beijing, China) was cultured with Dulbecco’s modified Eagle’s medium (DMEM, SH30022.01, HyClone, USA) containing 10% fetal bovine serum (FBS) at 37°C in a humidified atmosphere of 5% CO_2_. Afterward, BV2 cells were seeded in 6-well plates or 24-well plates and divided into four groups, defined as the NC group, IH group, HG (high glucose) group, and HG+IH group. When the cells reached 70% confluency, the NC group and the IH group were treated with 25 mM glucose as normal, and the HG group and the HG+IH group were treated with 50 mM glucose. For exposure to IH, the O_2_ concentration was decreased to 1.5% over 30 s and was then restored to 21% for 90 s (Song et al., 2017). The IH group and the HG+IH group were exposed to IH cycles for 8 h, and the NC group and the HG group were exposed to intermittent normoxic air cycles for 8 h.

### Enzyme-Linked Immunosorbent Assay (ELISA)

Blood was obtained from mice and centrifuged for 30 min at 4°C and 14,000 RPM. The supernatant was preserved at −80°C for subsequent analysis by ELISA. All ELISA steps were conducted following the manufacturer’s protocols.

### Immunofluorescent Labeling

The BV2 cell slides were fixed, permeated and blocked. Then, they were incubated with anti-NLRP3 (1:200, A5652, ABclonal Technology, Woburn, MA, USA) and anti-IL-1β (1:200, BS6067, Bioworld Technology, Inc., St Louis Park, MN, USA) antibodies at 4°C overnight. Next, the cells were incubated with fluorescein isothiocyanate (FITC) labeled anti-rabbit secondary antibody. After 60 min of the initial reaction, the cell nuclei were stained with 4,6-diamidino-2-phenylindole (DAPI, Beyotime, cat# C1002) for 60 s. Images were taken by a fluorescence microscope and analyzed using ImageJ software.

### Real-Time Quantitative Polymerase Chain Reaction (qRT-PCR)

Brain tissues and BV2 cells were dissolved with TRIzol reagent, and total RNA was first extracted according to the manufacturer’s protocol. Then, a reverse transcription kit (Invitrogen) was used to generate cDNA. RT-PCR analysis was carried out by SYBR Green PCR kits. Relative expression was calculated by the 2^−ΔΔCt^ method. Primer sequences are listed in Table 1.

**Table 1:** Primer sequences of RNAs.

RNAs	Primer sequence
m-NLRP3	F: ACACGAGTCCTGGTGACTTTG
	R: GGGCTTAGGTCCACACAGAAA
m-CASP1	F: AAGAATTTGCTGCCTGCCCA
	R: TCCACGGCATGCCTGAATAA
m-TNF-α	F: GCCTCTTCTCATTCCTGCTTGT
	R: GGCCATTTGGGAACTTCTCAT
m-IL-1β	F: TGCCACCTTTTGACAGTGATG
	R: AAGGTCCACGGGAAAGACAC
m-MALAT1	F: CAGTGCTGGGTGGGAATGTA
	R: TGGCCAAGTCTGTTATGTCCA
m-GAPDH	F: CATCACTGCCACCCAGAAGACTG
	R: ATGCCAGTGAGCTTCCCGTTCAG
m-224-5p	F: GGTCC TAAGTCACTAGTGGTTCCGTT
m-216-5p	F: GGTCC AAATCTCTGCAGGCAAATGTGA
m-485-5p	F: GGTCC AGAGGCTGGCCGTGATGAATTC
Universal reverse primer	R: CCAGTGCAGGGTCCGAGGT
m-U6	F: CGCACTTTACGGCTACCTCT
	R: CGCCCCAGACTGAAAAAGAC

### Immunohistochemistry (IHC)

Hippocampal paraffin sections included in cortical tissues were mounted, dewaxed, hydrated, and rinsed in PBS 3 times. The immunohistochemistry (IHC) method used in this study was as described in C.A. Oyinbo’s study (Oyinbo et al., 2018). The sections were boiled in 0.01 M sodium citrate hydrochloric acid (pH = 6.0) for 20 min. After blocking with 0.03% H_2_O_2_ at room temperature and then washing three times with PBS, the slides were also blocked in 1% BSA for 1 h. After washing again, the slides were incubated with goat anti-rabbit NLRP3 (1:200, A5652, ABclonal Technology, Woburn, MA, USA) antibody diluted in 1% BSA overnight at 4°C. The sections were incubated in HRP-conjugated anti-rabbit secondary antibody at a dilution of 1:5,000 for 30 min, followed by washing with PBS. Then, they were incubated in a chromogen solution in 3,3′-diaminobenzylamine (DAB, Dako Cytomation, CA, USA) until a light brown color appeared on the slide. Finally, they were counterstained with hematoxylin, mounted, and examined on a microscope.

### Firefly Luciferase Assay

BV2 cells were seeded on a 24-well plate at a density of approximately 5 × 10^4^ cells/well for 24 h before transfection (Sutliff et al., 2019). Then, they were transfected with a plasmid containing the firefly luciferase gene, which has a complementary miR-224-5p binding site in its 3′ untranslated region (UTR) and was purchased from GenePharma (Shanghai, China; Sureban et al., 2011). Luciferase activity is expressed in relative light units (RLU).

### Cell Transfection

We constructed an overexpression vector (MALAT1) that amplified the MALAT1 sequence and used a mutated vector (vector) as a control (Xu et al., 2011). The overexpression and inhibition of miR-224-5p were achieved by miR-224-5p mimics and miR-224-5p inhibitors, with nontargeting control (NC) mimics and nontargeting control (NC) inhibitor as controls. To transfect BV2 cells, the miR-224-5p mimic and miR-224-5p inhibitor were synthesized by GenePharma (Shanghai, China) as follows: miR-224-5p mimic sense: 5′-UAAGUCACUAGUGGUUCCGUU-3′; antisense: 5′-UUAUUCAGUGAUCACCAAGGC-3′; miR-224-5p inhibitor: 5′-AACGGAACCACUAGUGACUUA-3′. NC sense: 5′-UUCUCCGAACGUGUCACGUTT-3′; antisense: 5′-ACGUGACACGUUCGGAGAATT-3′. BV2 cells were seeded at 1 × 10^5^ cells/well in a 6-well plate at 37°C in a 5% CO_2_ incubator. When cells reached 70% confluency the next day, they were transfected with the MALAT1 vector, control vector, NC mimics, NC inhibitor, miR-224-5p mimics or miR-224-5p inhibitor. The transfection was carried out with Lipofectamine 2000 (Invitrogen, USA) according to the manufacturer’s protocol.

### Western Blotting

We performed western blot assays to detect the expression of NLRP3, caspase 1, TNF-α, and IL-1β. Cells or brain tissues were lysed with radioimmunoprecipitation assay buffer (RIPA, Beyotime, China) supplemented with PMSF, phosphatase inhibitors, and loading buffer. An equal amount of protein was further separated by 10% SDS-PAGE and then transferred to a polyvinylidene fluoride (PVDF) membrane by semi-dry transfer. The membranes were incubated with Tris-buffered saline-Tween (TBST) containing 5% fat-free milk for 2 h to avoid non-specific binding. Then, the primary antibodies against NLRP3 (1:1,000, A5652, ABclonal Technology, Woburn, MA, USA), caspase 1 (1:1,000, 22915–1-AP, Proteintech Group, Rosemont, IL, USA), TNF-α (1:500, BS1857, Bioworld Technology, St Louis Park, MN, USA), IL-1β (1:500, 16806-1-AP, Proteintech Group, Rosemont, IL, USA) and GAPDH (1:5,000, AP0063, Bioworld Technology, St Louis Park, MN, USA), were incubated at 4°C overnight. After rinsing with buffer, the membranes were incubated with horseradish peroxidase-conjugated anti-rabbit antibody (1:5,000 dilution) at room temperature for 1 h. After washing, chemiluminescence signals in the membranes were developed by ECL Blotting Detection Reagents (WBKLS0500, Millipore Corporation, Burlington, MA, USA), and blots were quantified with ImageJ software.

### Statistical Analysis

Data are expressed as the mean ± SD. Comparisons between more than two groups were analyzed by one-way ANOVA followed by the Tukey post-test. *P* < 0.05 was considered to be statistically significant.

## Results

### The Inflammatory Response Was Significantly Enhanced in T2DM Mice Exposed to IH

**Figure 1:**
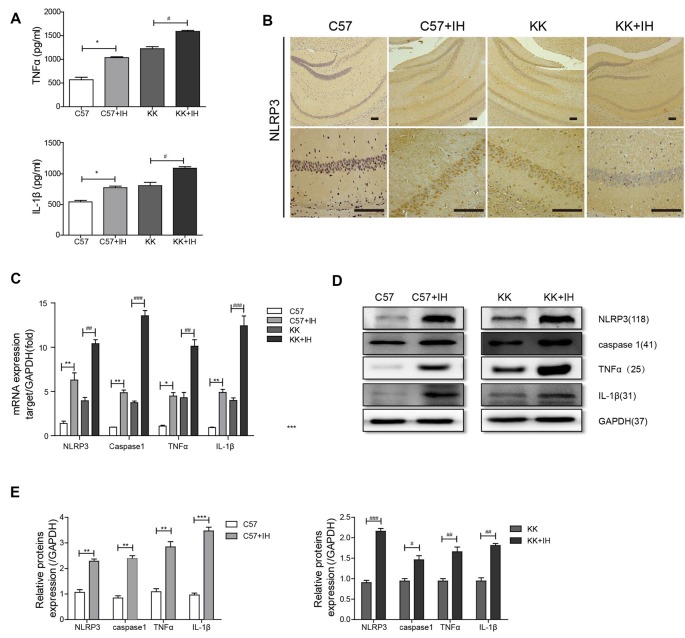
The inflammatory response in the brain was increased in type 2 diabetes mellitus (T2DM) mice exposed to intermittent hypoxia (IH). **(A)** Tumor necrosis factor-α (TNF-α) and interleukin-1 β (IL-1β) in mouse serum were measured by enzyme-linked immunosorbent assay (ELISA). **(B)** NLRP3 expression was determined by immunohistochemistry (IHC). Scale bars in the right lower corner represent 20 mm. **(C)** NLRP3, caspase 1, TNF-α and IL-1β levels were determined by Real-time quantitative polymerase chain reaction (qRT-PCR). **(D,E)** NLRP3, caspase 1, TNF-α and IL-1β levels were determined by western blot. The data are presented as the mean ± SEM. *p < 0.05, **p < 0.01, ***p < 0.001 vs. the C57 group; ^#^*p* < 0.05, ^##^*p* < 0.01, ^###^*p* < 0.001 vs. the KK+IH group.

We measured TNF-α and IL-1β in mouse serum by ELISA (Figure 1A). The results showed that TNF-α and IL-1β both increased in the C57+IH group and the KK group compared to the C57 control group. Additionally, they were most increased in the KK+IH group. We discovered that the expression of NLRP3 in the C57+IH group and the KK group was significantly higher than that in the C57 normal group by IHC. The expression of NLRP3 in the KK+IH group was significantly increased compared with that in the other groups (Figure 1B). The mRNA expression levels of NLRP3, caspase 1, TNF-α and IL-1β in brain tissues of the different groups were analyzed by qRT-PCR. There was a significantly higher mRNA level of NLRP3, caspase 1, TNF-α and IL-1β in brain tissues of the KK+IH group (Figure 1C). The protein levels of NLRP3, caspase 1, TNF-α and IL-1β in the tissues of the C57+IH group and the KK group were higher than those of the C57 group, and the protein expression of these inflammatory factors was the highest in the KK+IH group (Figures 1D,E).

### IH Increased the Expression of MALAT1 and Decreased the Expression of miRNAs in T2DM Mice

**Figure 2:**
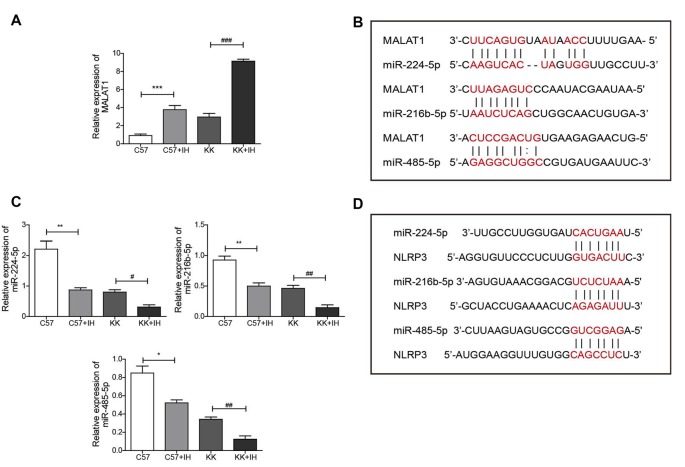
IH upregulated MALAT1 and downregulated miRNAs in the brains of T2DM mice. **(A,C)** MALAT1 and the miRNAs miR-224-5p, miR-216b-5p and miR-485-5p were analyzed by qRT-PCR. **(B)** Base-pairing sites for MALAT1 and miR-224-5p, miR-216b-5p and miR-485-5p. **(D)** Base-pairing sites for miR-224-5p, miR-216b-5p and miR-485-5p with NLRP3. All results are presented as the mean ± SEM. *p < 0.05, **p < 0.01, ***p < 0.001 vs. the C57 group; ^#^*p* < 0.05, ^##^*p* < 0.01, ^###^*p* < 0.001 vs. the KK+IH group.

To reveal the roles of MALAT1, NLRP3 and miR-224-5p and their relationship in the T2DM with the OSA model, the expression of MALAT1 and miR-224-5p in normal C57 mice, C57 mice exposed to IH, KKAy mice and KKAy mice exposed to IH was analyzed by qRT-PCR. The results showed that MALAT1 was upregulated in the C57+IH group compared with the C57 group. The expression level of MALAT1 was greatly increased in the KK+IH group than in the KK group (Figure 2A). We found base-pairing sites for MALAT1 and miR-224-5p, miR-216b-5p and miR-485-5p *via* the TargetScan website (Figure 2B). Conversely, miR-224-5p, miR-216b-5p and miR-485-5p were significantly down-regulated in the KK+IH group than these in the C57 group (Figure 2C). We found base-pairing sites for miR-224-5p, miR-216b-5p and miR-485-5p with NLRP3 *via* the StarBase database (Figure 2D). Therefore, we predicted that MALAT1 competitively bound to miR-224-5p to block the inhibition of NLRP3 expression mediated by miR-224-5p.

### MALAT1 and NLRP3 Were Highly Expressed and miR-224-5p Was Downregulated in BV2 Cells Exposed to IH and HG

**Figure 3:**
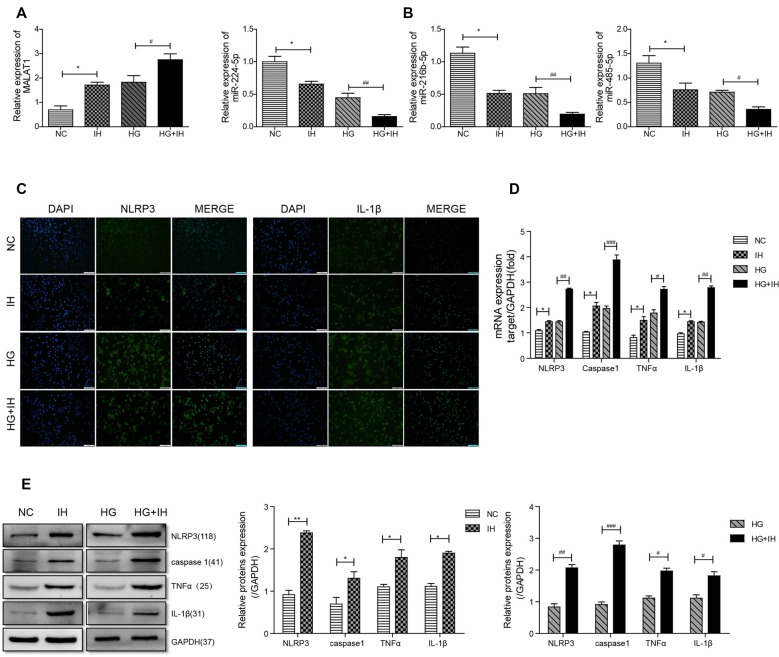
HG (high glucose) combined with IH aggravated the activation of BV2 microglial cells and upregulated MALAT1 and downregulated miRNAs. **(A,B)** MALAT1 and the miRNAs miR-224-5p, miR-216b-5p and miR-485-5p were analyzed by qRT-PCR. **(C)** Immunofluorescence was used to analyze the expression of NLRP3 and IL-1β in each group of BV2 cells. **(D,E**) qRT-PCR and western blot were used to analyze NLRP3, caspase 1, TNF-α and IL-1β expression levels in BV2 cells. *p < 0.05, **p < 0.01 vs. the NC group; ^#^*p* < 0.05, ^##^*p* < 0.01, ^###^*p* < 0.001 vs. the HG+IH group.

We first observed by qRT-PCR that, compared with that in the NC group, the expression level of MALAT1 was increased in the IH group and the HG group, and the expression in the HG+IH group was the most obviously increased (Figure 3A). Conversely, the expression levels of miR-224-5p, miR-216b-5p and miR-485-5p in BV2 cells were decreased in both the HG group and the IH group compared with the NC group. The reductions in the expression levels of these miRNAs were greater in the HG+IH group than in the HG group (Figure 3B). Immunofluorescence was used to analyze the expression of inflammatory factors in each group of BV2 cells. We found that the expression of NLRP3 and IL-1β was significantly increased in the IH group and the HG group compared with the NC group, and these levels were most increased in the HG+IH group compared with the other groups (Figure 3C). As with the tissue results, the mRNA expression levels of NLRP3, caspase 1, TNF-α, and IL-1β were higher in BV2 cells exposed to IH or HG than in the normal control group, and the levels in BV2 cells exposed to IH and HG were significantly higher than those in BV2 cells exposed to IH or HG (Figure 3D). We verified the protein expression levels by western blotting. The results showed that the expression of NLRP3, caspase 1, TNF-α and IL-1β was upregulated in the IH group, HG group and HG + IH group, while it was most prominent in the IH+HG group (Figure 3E).

### Inflammation Is Affected by Exposure to IH and HG *via* the MALAT1/miR-224-5p/NLRP3 Axis

**Figure 4:**
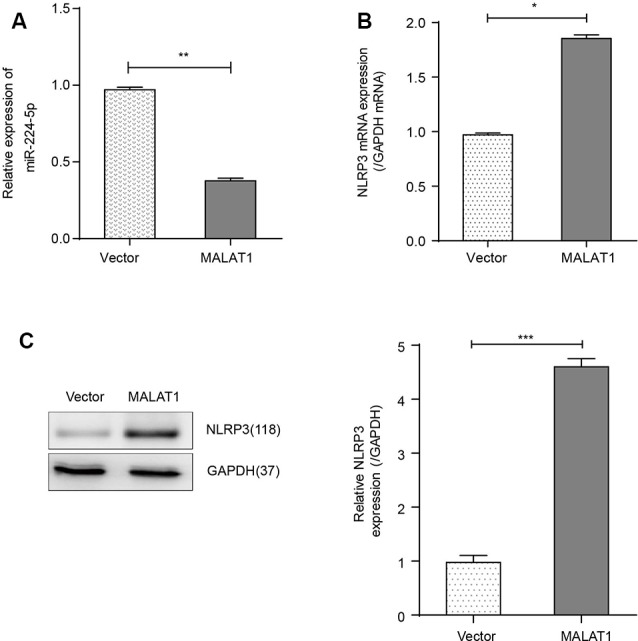
Overexpression of MALAT1 promoted the inflammatory response of BV2 cells. **(A)** The results of qRT-PCR showed that the expression of miR-224-5p in MALAT1-transfected BV2 cells decreased significantly compared with that in control (vector) BV2 cells. **(B)** qRT-PCR results showed that the mRNA expression of NLRP3 was higher in MALAT1-overexpressing BV2 cells compared with control (vector) BV2 cells. **(C)** The results of western blot analysis showed that the expression of NLRP3 was upregulated in MALAT1-overexpressing BV2 cells compared with control (vector) BV2 cells. *p < 0.05, **p < 0.01, ***p < 0.001 vs. the vector group.

Overexpression of MALAT1 in BV2 cells significantly reduced the expression of miR-224-5p (Figure 4A) while increasing the expression of NLRP3 (Figures 4B,C).

**Figure 5:**
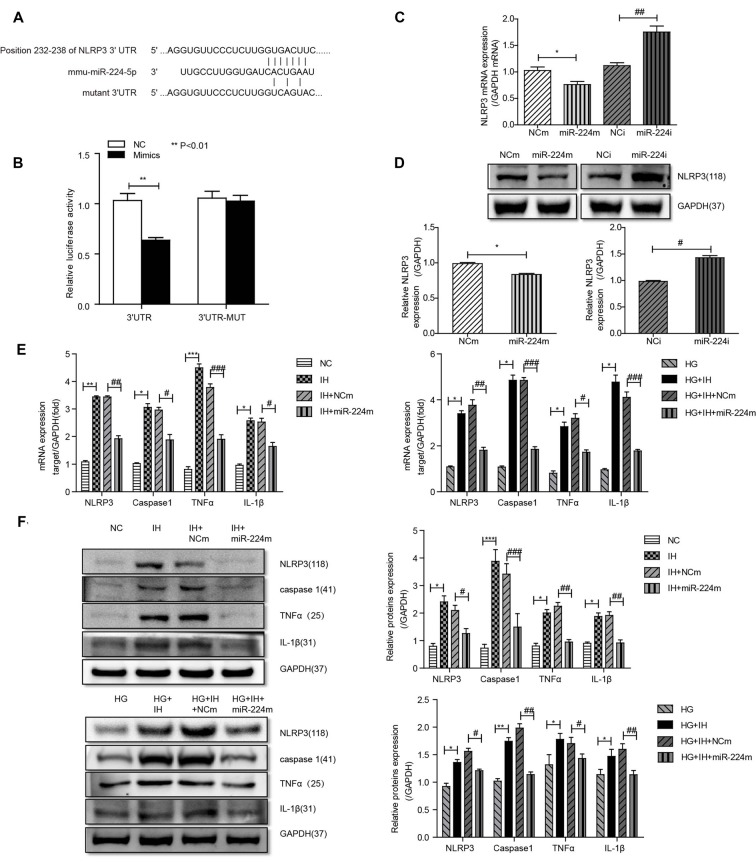
Inhibition of the MALAT1/miR-224-5p/NLRP3 axis reduced inflammation caused by exposure to IH and HG. **(A)** Sequence alignment between miR-224-5p and the 3′-untranslated region (UTR) of NLRP3. Complementary bases between the sequences are shown in red. The sequence of the mutant NLRP3 construct is also shown. **(B)** Firefly luciferase assay of BV2 cells co-transfected with NLRP3 3′-UTR-WT or NLRP3 3′-UTR-Mut and miR-224-5p mimic or miR-NC. Data are presented as the mean ± SD from six separate experiments. **p < 0.01. **(C,D)** After the transfection of mimics and inhibitor, the mRNA expression and protein expression of NLRP3 was determined by qRT-PCR **(C)** and western blotting **(D)**. **(E,F)** NLRP3, caspase 1, TNF-α and IL-1β expression levels in different groups of BV2 cells were detected by qRT-PCR **(E)** and western blotting **(F)**. *p < 0.05, **p < 0.01, ***p < 0.001 vs. the NC group; ^#^*p* < 0.05, ^##^*p* < 0.01, ^###^*p* < 0.001 vs. the HG+IH+miR-224m group.

Alignment of the miR-224-5p sequence and the 3′-UTR of NLRP3 indicated complementary regions constituting a putative miR-224-5p target site in the NLRP3 gene (Figure 5A). A dual-luciferase reporter assay was performed to assess the interaction between miR-224-5p and the 3′-UTR of NLRP3. Co-transfection of BV2 cells with NLRP3 3′-UTR-WT and the miR-224-5p mimic resulted in significantly reduced luciferase activity compared with the miR-NC construct (*P* < 0.01; Figure 5B). When the NLRP3 3′-UTR-mut was co-transfected, no significant difference was seen between the miR-224-5p mimic and miR-NC construct, confirming the specific interaction between miR-224-5p and the 3′-UTR of NLRP3.

**Figure 6:**
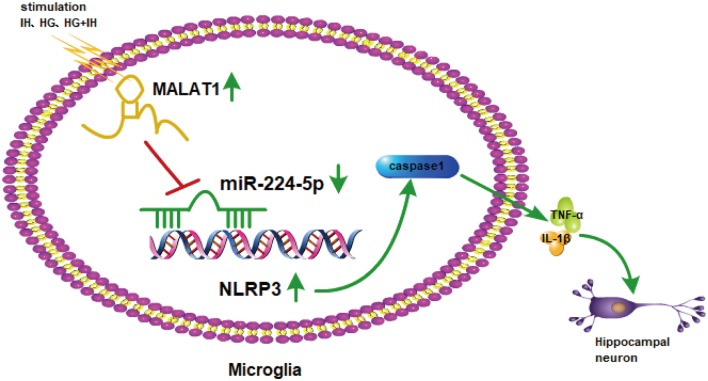
The mechanism involved in hippocampal neuronal apoptosis induced by the MALAT1/miR-224-5p/NLRP3 axis after stimulation by HG combined with IH.

Previous results suggested that the upregulation of miR-224-5p may inhibit the inflammatory response of BV2 cells. To further investigate the function of miR-224-5p in the activation and inflammatory response of BV2 cells *in vitro*, a transfection experiment was performed. After the transfection of mimics and inhibitor, the knockdown efficiency of miR-224-5p was confirmed by qRT-PCR (Figure 5C) and western blotting (Figure 5D). The mRNA expression and the protein expression of NLRP3 in BV2 cells were tested. It was found that miR-224-5p overexpression significantly reduced the mRNA and protein expression of NLRP3 in BV2 cells, while inhibition of miR-224-5p improved their expression. The western blot results showed that the expression level of NLRP3 increased with the decrease in miR-224-5p, which also agreed with the prediction analysis results of the StarBase database. To discover the effects of miR-224-5p mimics on BV2 cell activation, transfection was performed in cells under different treatment conditions. qRT-PCR and western blotting results indicated that miR-224-5p mimics inhibited the expression of NLRP3, caspase 1, TNF-α and IL-1β in BV2 cells of the different groups. We found that at the mRNA levels of NLRP3, caspase 1, TNF-α and IL-1β were downregulated in the group transfected with miR-224-5p mimics after exposure to IH than the negative control group and the IH exposure group. Similarly, mRNA expression levels of NLRP3, caspase 1, TNF-α and IL-1β were lower in the group transfected with miR-224-5p mimics after exposure to IH+HG than in the negative control group and the IH+HG exposure group (Figure 5E). Furthermore, we also validated the results at the protein level. Transfection of the miR-224-5p mimics reduced the protein expression level of inflammatory factors under the same exposure conditions (Figure 5F). Taken together, our findings indicate that hippocampal neuronal apoptosis induced by the MALAT1/miR-224-5p/NLRP3 axis after stimulation by HG combined with IH (Figure 6).

## Discussion

One of the main concerns in recent years has been the overall increase in the incidence associated with T2DM, including OSA (Leow, 2010). Studies have shown that individuals with T2DM can develop memory and cognitive dysfunction, which greatly affects the quality of life of affected populations (Gispen and Biessels, 2000). Chronic intermittent hypoxia is a major consequence of OSA and is associated with cognitive dysfunction (Xu et al., 2020). Our previous studies have shown that intermittent hypoxia can aggravate cognitive dysfunction in T2DM by neuroinflammation-induced apoptosis in hippocampal neurons (Shi et al., 2018). Hypoxic cell death is associated with inflammatory processes triggered by activated microglia (Slowik et al., 2018). Interestingly, inflammatory factors in serum, including TNF-α and IL-1β, were increased in KKAy mice and C57 mice exposed to IH, while they were significantly greater in KKAy mice exposed to IH. Also, we found that the expression level of NLRP3 in IHC was completely consistent with the ELISA results. The mRNA and protein expression levels of NLRP3, caspase 1, TNF-α and IL-1β were upregulated in KKAy mice and C57 mice exposed to IH, and these factors had the highest expression in KKAy mice exposed to IH. Our results demonstrated that IH aggravates the brain inflammatory response in patients with T2DM. In the normal central nervous system (CNS), microglia play an important role in maintaining the balance of neuron differentiation and apoptosis by producing neurotrophic proteins (Zhao et al., 2019). There is evidence that several miRNAs are important transcriptional regulators of certain inflammation-related mediators (Marques-Rocha et al., 2015). In this study, we investigated the role of miRNAs in the development of microglial inflammation. We chose the BV2 cell line as the microglial cell line. Our results showed that HG or HG+IH treatment induced an increase in the mRNA and protein expression levels of NLRP3, caspase 1, TNF-α and IL-1β.

Many relevant studies have indicated that lncRNAs have a significant role in the occurrence and development of many diseases (Chen et al., 2018), such as chronic inflammation-related diseases. MALAT1 affects many biological processes, including cell proliferation, migration, and apoptosis (Li et al., 2017). Studies have shown that MALAT1 is primarily located in the cytoplasm, indicating that it is a post-transcriptional regulator in cells (Chen et al., 2018). Our results showed that the expression trend of MALAT1 was consistent with the expression trend of inflammatory factors. Thus, we reasonably conclude that MALAT1 may promote inflammation of the brain. In our study, we obtained consistent results in tissue experiments and cell experiments, thus confirming our hypothesis.

Research has demonstrated that MALAT1 sponges miR-22, decreases miR-22 expression and thus modulates pyroptosis (Song et al., 2019). Additionally, MALAT1 was found to promote the angiogenesis of cerebral microvascular endothelial cells by targeting miR-145 (Ren et al., 2019). MALAT1 could directly bind miR-224-5p, miR-216b-5p and miR-485-5p and there was an effect on those miRNA expression levels. In this experiment, we found that MALAT1 could downregulate the expression of those miRNAs, among which miR-224-5p was the most obviously changed, so we chose miR-224-5p for subsequent research. miR-224-5p plays a role in many diseases, such as hepatocellular carcinoma (Li J. et al., 2019), glioma (Zheng et al., 2019) and Alzheimer’s disease (Zhu L. et al., 2019). Additionally, the function of miR-224-5p in T2DM and OSA has not been investigated. Luciferase assays confirmed that miR-224-5p, as a target of MALAT1, directly reduced the expression of the downstream protein NLRP3. In the present study, miR-224-5p overexpression significantly inhibited inflammation *in vitro*. Importantly, qRT-PCR and western blotting were performed to reveal the underlying mechanisms of miR-224-5p. Regarding T2DM and OSA, we demonstrated that miR-224-5p was the most downregulated miRNA in the brains of KKAy mice exposed to IH, and *in vitro* microglial cells exposed to HG+IH had low miR-224-5p expression levels compared to microglial cells in the control group. The expression level of miR-224-5p was decreased in MALAT1-upregulated BV2 cells.

A study revealed that lncRNA MALAT1 affected the progression of CAD in EPC autophagy by regulating miR-15b-5p and its target genes in the MAPK1 and mTOR pathways (Zhu Y. et al., 2019). Interestingly, although MALAT1 does not bind with NLRP3 mRNA directly, MALAT1 and NLRP3 mRNA contain the same putative miR-224-5p binding sequence. In the present study, we discovered that MALAT1 and NLRP3 mRNA shared the same miR-224-5P binding sites. Also, we confirmed that there was a reciprocal inhibition effect between MALAT1 and miR-224-5p. In our study, we specifically focused on the role of MALAT1 in the regulation of miR-224-5p and its downstream target, NLRP3. We have shown that MALAT1 overexpression was positively related to NLRP3 protein expression and negatively related to miR-224-5p expression, suggesting that there was a regulatory network in microglial cells. Furthermore, we demonstrated that overexpression of MALAT1 promoted the inflammatory response of BV2 cells.

Activation of the microglial NLRP3 inflammasome is becoming a key factor in neuroinflammation during neurodegenerative processes (Deora et al., 2020). The NLRP3 inflammasome is a kind of macromolecular multiprotein complex with a molecular weight of ~700 kDa, which consists of NLRP3, adaptor protein ASC and effector protein caspase 1 (Martinon et al., 2002). Activated NLRP3 recruits the downstream proteins ASC and caspase 1 to form the NLRP3 inflammasome as a core protein (Slowik et al., 2018). Research has revealed that NLRP3 can increase the expression of IL-1β by activating the NF-κB signaling pathway and further enhance the effect of the NLRP3 inflammasome (Zhang et al., 2019). The competitive binding of MALAT1 and miR-224-5p resulted in the upregulation of NLRP3, which led to the inflammatory activation of microglia and neurotoxicity of neurons. Our study focuses on miR-224-5p, which plays a key role in the maturation of IL-1β through the inhibition of the NLRP3 inflammasome-caspase 1 complex. To investigate whether the protective function of miR-224-5p is related to the NLRP3/IL-1β signaling pathway, the expression levels of NLRP3, caspase 1, TNF-α and IL-1β in BV2 cells were examined. It was determined that the expression levels of all four proteins were significantly enhanced in the miR-224-5p inhibitor group compared with the NC group, while the expression levels of the above proteins were decreased following miR-224-5p mimics transfection. This indicated that the protective effects of miR-224-5p against NLRP3 may be partially mediated by inhibiting the activation of the NLRP3 inflammasome.

## Conclusion

In conclusion, our work demonstrated that MALAT1 and NLRP3 were overexpressed in KKAy mice exposed to IH and microglial cells exposed to HG+IH, which was negatively correlated with the expression of miR-224-5p. MiR-224-5p reduced microglial inflammation activation by regulating NLRP3 expression, which finally regulated the NLRP3/IL-1β pathway in the hippocampus. This suggests that miR-224-5p may serve as a potential target for T2DM with OSA therapy.

## Data Availability Statement

The author’s raw data supporting the article’s conclusions will be made available to any qualified researcher without reservation.

## Ethics Statement

The animal study was reviewed and approved by The Animal Ethics Committee of Tianjin Medical University.

## Author Contributions

PD and JW performed all experiments, analyzed data and wrote the manuscript. YH edited the manuscript. JF designed the study, directed and supervised the study. All authors have endorsed the final version of the manuscript.

## Conflict of Interest

The authors declare that the research was conducted in the absence of any commercial or financial relationships that could be construed as a potential conflict of interest.
